# Additional chemoradiotherapy for superficial esophageal squamous cell carcinoma after near-circumferential or full-circumferential noncurative endoscopic submucosal dissection: a retrospective study

**DOI:** 10.1186/s12876-024-03328-2

**Published:** 2024-07-23

**Authors:** Yutaro Tasaki, Takuya Yamazaki, Shuhei Miyazaki, Tatsuya Takeda, Mika Nakatake, Daisuke Nakamura, Asuka Takahira, Koichi Honda, Akiko Egawa, Naoyuki Yamaguchi, Kazuhiko Nakao, Ryo Toya

**Affiliations:** 1 Department of Radiology, Nagasaki University Hospital, 1-7-1 Sakamoto, Nagasaki, 852-8501 Japan; 2 Department of Radiology, Sasebo Central Hospital, 15 Daiwa, Sasebo, 857-1195 Japan; 3 Department of Radiology, National Hospital Organization Nagasaki Medical Center, 1000-1 Kubara, Omura, 856- 8562 Japan; 4 Department of Radiology, Japanese Red Cross Nagasaki Genbaku Hospital, 3-15 Mori, Nagasaki, 852-8511 Japan; 5 Department of Clinical Oncology, Nagasaki University Hospital, 1-7-1 Sakamoto, Nagasaki, 852-8501 Japan; 6 Department of Gastroenterology and Hepatology, Nagasaki University Hospital, 1-7-1 Sakamoto, Nagasaki, 852- 8501 Japan

**Keywords:** Chemoradiotherapy, Esophageal cancer, Endoscopic submucosal dissection, Muscularis mucosae

## Abstract

**Background:**

Endoscopic submucosal dissection (ESD) is a potentially efficient therapeutic intervention for superficial esophageal cancer. Additional treatment such as chemoradiotherapy (CRT) or esophagectomy is recommended in cases of muscularis mucosa invasion with positive resection margins or lymphovascular invasion or submucosal layer invasion, which are considered noncurative ESD, due to an increased risk of lymph node metastasis. However, the adequacy of additional CRT after near-circumferential or full-circumferential noncurative ESD has not been fully discussed. In this study, we retrospectively evaluated the efficacy and toxicity of additional CRT for superficial esophageal squamous cell carcinoma (SCC) after near-circumferential or full-circumferential noncurative ESD, which was defined as a mucosal defect measuring ≥ 3/4 of the esophageal circumference.

**Methods:**

We retrospectively evaluated 24 patients who received additional CRT for superficial esophageal SCC after near-circumferential or full-circumferential noncurative ESD between 2012 and 2018. Elective nodal irradiation (ENI) was performed in all patients and boost irradiation (BI) was performed after ENI in 4 patients with positive resection margins. The prescription doses of ENI and BI were 41.4 Gy in 23 fractions and 9 Gy in 5 fractions, respectively. Concurrent chemotherapy (a combination of cisplatin or nedaplatin and 5-fluorouracil) was administered to all patients.

**Results:**

The 3-year and 5-year overall survival rates were 92% and 78%, respectively, while the 3-year and 5-year progression-free survival rates were 83% and 70%, respectively. Grade 2 esophageal stenosis occurred in 8 (33%) patients. There was no case of Grade 3 or worse esophageal stenosis. Among them, 4 (17%) patients developed stenosis before additional CRT, which persisted after the completion of additional CRT. The remaining 4 (17%) patients developed *de novo* stenosis within 5 months following the completion of additional CRT. One patient (4%) still requires regular bougie dilation. Grade 3 and Grade 4 acute toxicity, including anemia, neutropenia, thrombocytopenia, and esophagitis occurred in 1 (4%) and 0 (0%), 6 (25%) and 1 (4%), 1 (4%) and 0 (0%), and 1 (4%) and 0 (0%) patients, respectively. One (4%) patient who underwent salvage CRT for the out-of-field lymph node recurrence died with acute myeloid leukemia.

**Conclusions:**

Additional CRT is a viable treatment option even in patients who have undergone near-circumferential or full-circumferential noncurative ESD. Esophageal stenosis after additional CRT following near-circumferential or full-circumferential noncurative ESD is manageable and acceptable.

## Background

Recently, owing to advancements in endoscopic cancer screening technology, early-stage esophageal cancer detection rates have increased [1, 2]. Endoscopic submucosal dissection (ESD) is a potentially efficient curative intervention for superficial esophageal cancer. However, in patients with muscularis mucosae (MM) or more profound invasion, there is an increased risk of lymph node metastasis. Lymph node metastasis occurs in 10–20% of cases of MM or upper submucosal layer (SM1) invasion and 40–60% of the middle submucosal layer (SM2) or lower submucosal layer (SM3) invasion [3, 4]. Additional treatment such as chemoradiotherapy (CRT) or esophagectomy is recommended in cases of MM invasion with positive resection margins or lymphovascular invasion (LVI) or SM invasion, which are classified as noncurative ESD [5]. Recent studies on superficial esophageal cancer have reported that survival outcomes of definitive CRT are comparable with those of esophagectomy; increasing attention has been given to the combination of ESD and CRT as a minimally invasive organ-preservation strategy, unlike esophagectomy [6]. However, the combination of ESD and additional CRT is commonly performed for limited primary tumors [7], and the adequacy of additional CRT after near-circumferential or full-circumferential noncurative ESD has not been fully discussed. In this study, we retrospectively evaluated the efficacy and toxicity of additional CRT for superficial esophageal squamous cell carcinoma (SCC) after near-circumferential or full-circumferential noncurative ESD, which was defined as the presence of mucosal defects measuring ≥ 3/4 of the esophageal circumference.

## Methods

### Patients

**Fig. 1:**
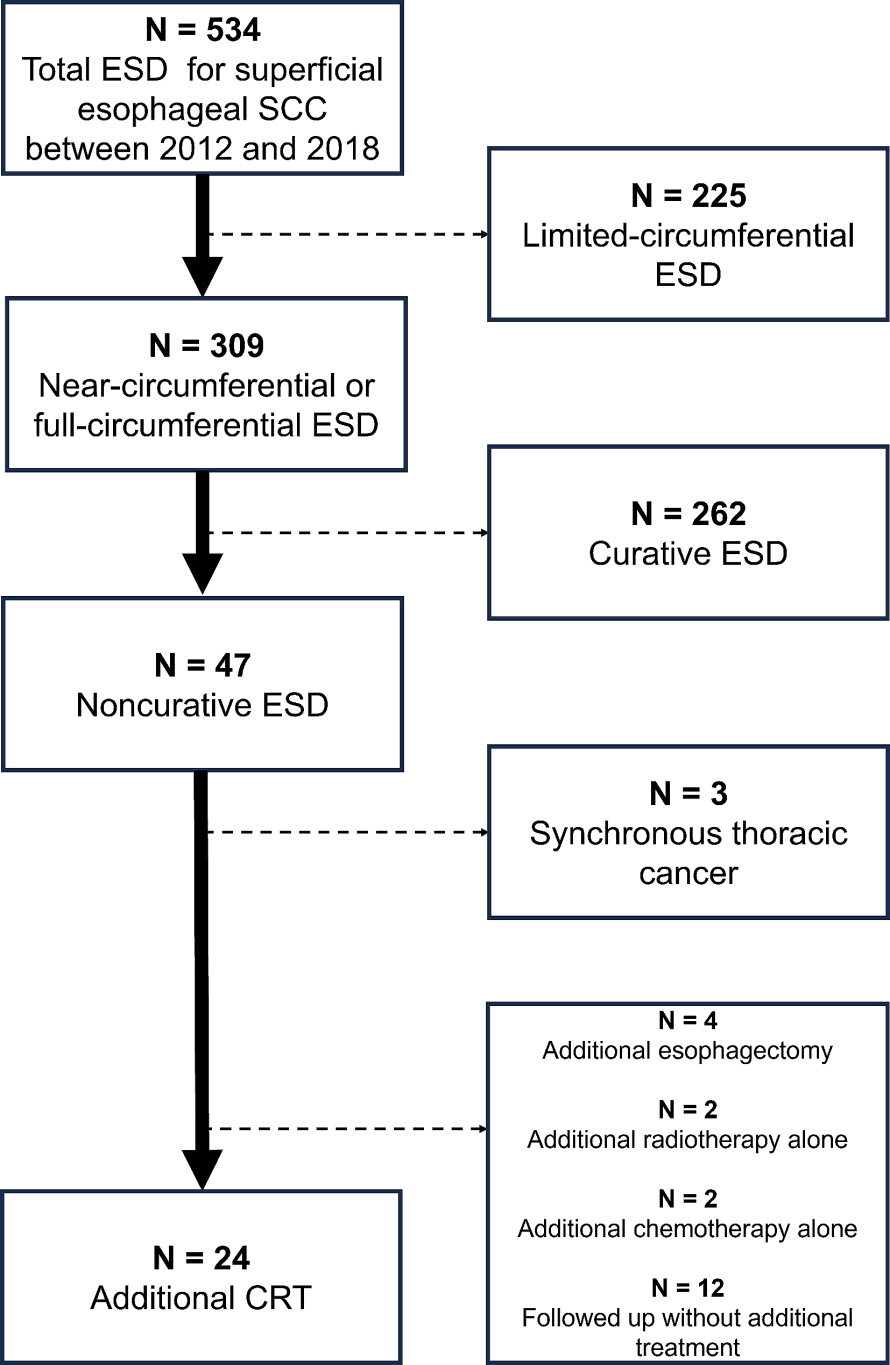
Flow diagram showing the selection process

This retrospective analysis was approved by the institutional review board (No.20,061,507) of Nagasaki University Hospital. Figure 1 summarizes the selection process of the patients. Between 2012 and 2018, we performed ESD for superficial esophageal SCC in consecutive 534 patients. The inclusion criteria were as follows: (1) histologically proven esophageal SCC, (2) near-circumferential or full-circumferential ESD, which was defined as the presence of mucosal defects measuring ≥ 3/4 of the esophageal circumference, (3) pathological noncurative ESD, which includes MM invasion with positive resection margins or LVI or SM invasion, (4) clinically node-negative (cN0) and no metastasis to other organs (cM0), (5) absence of synchronous thoracic cancer, and (6) received additional CRT [7]. Of the 47 patients with noncurative ESD, 4 underwent additional esophagectomy, 2 received additional radiotherapy (RT) alone, 2 received additional chemotherapy alone, and 12 were followed up without additional treatment. The decision between CRT and esophagectomy was made based on patient preference after explanation by radiation oncologists and gastrointestinal surgeons. Of the 2 patients who received radiotherapy alone, one refused esophagectomy and chemotherapy, while other was unsuitable for both due to liver dysfunction. Two patients who received chemotherapy alone refused esophagectomy, and radiotherapy was contraindicated due to history of thoracic irradiation. Among the 12 patients who were followed without additional treatment, 7 declined further treatment based on their preferences, while the remaining 5 were considered ineligible for additional therapy owing to poor performance status, advanced age or organ dysfunction.

### Treatment

Written informed consent was obtained from all patients before treatment initiation. After ESD, all patients received prophylaxis for stenosis (oral prednisolone in 4 patients, intralesional steroid injection in 9 patients, a combination of oral prednisolone and steroid injection in 8 patients, and endoscopic transplantation of tissue-engineered cell sheets in 3 patients) [8]. Prior to conducting a planning computed tomography (CT), metallic clips were endoscopically placed to mark the excision region. CT simulation was performed with a dedicated pillow for RT [9]. The arms of the patients were not elevated above their heads. The clinical target volume (CTV) for the primary lesion (CTV-p) was defined as an area of excision region with a 2-cm margin in the longitudinal direction. The clinical target volumes for the subclinical region (CTV-s) according to the primary tumor sites were supraclavicular, upper mediastinal, and subcarinal lymph node areas for cervical and upper thoracic tumors, upper-to-lower mediastinal, and perigastric lymph node areas for middle thoracic tumors, and middle-to-lower mediastinal, perigastric, and celiac artery lymph node areas for lower thoracic tumors [7]. The planning target volumes for primary tumor (PTV-p) and for subclinical regional lymph node areas (PTV-s) were defined as CTV-s with 0.5-cm margins. Elective nodal irradiation (ENI) was performed for PTV-p and PTV-s in all patients and boost irradiation (BI) was performed for PTV-p after ENI in 4 patients with positive resection margins. The prescription doses of ENI and BI were 41.4 Gy in 23 fractions and 9 Gy in 5 fractions, respectively. Patients underwent additional CRT, with a median period of 96 days (range: 35–200 days) from ESD to the start of additional CRT. RT was administered as three-dimensional conformal RT (3D-CRT).

Concurrent chemotherapy (a combination of cisplatin (CDDP) or nedaplatin (NDP) and 5-fluorouracil (5-FU)) was administered to all patients. The use of chemotherapy regimens was determined based on each patient’s renal function. Fifteen patients received two cycles of CDDP (70 mg/m^2^/day on day 1) and 5-FU (700 mg/m^2^/day on days 1–4) at 4–5-week intervals and 2 patients received one cycle of the same regimen. Seven patients received two cycles of NDP (100 mg/m^2^/day on day 1) and 5-FU (700 mg/m^2^/day on days 1–4) at 4–6-week intervals.

### Follow-up

Patients were monitored at 4–6-month intervals; endoscopy was performed during each follow-up visit, and CT imaging with or without [^18^F] fluorodeoxyglucose positron emission tomography/CT (^18^F-FDG PET/CT) imaging was performed annually. Toxicity was assessed per the National Cancer Institute Common Toxicity Criteria ver. 5.0.

### Statistical analysis

Overall survival (OS) and progression-free survival (PFS) were calculated from the initiation of additional CRT. Progression was defined as local recurrence, lymph node metastasis, and/or distant metastasis. Local recurrence was defined as a neoplastic lesion detected at the site of the ESD scar [10]. Survival rates were determined using the Kaplan–Meier method. Univariate analyses were performed using the Log-rank test to identify potential determinants of OS and PFS. P-values of < 0.05 were considered statistically significant.

## Results

### Survival

**Fig. 2:**
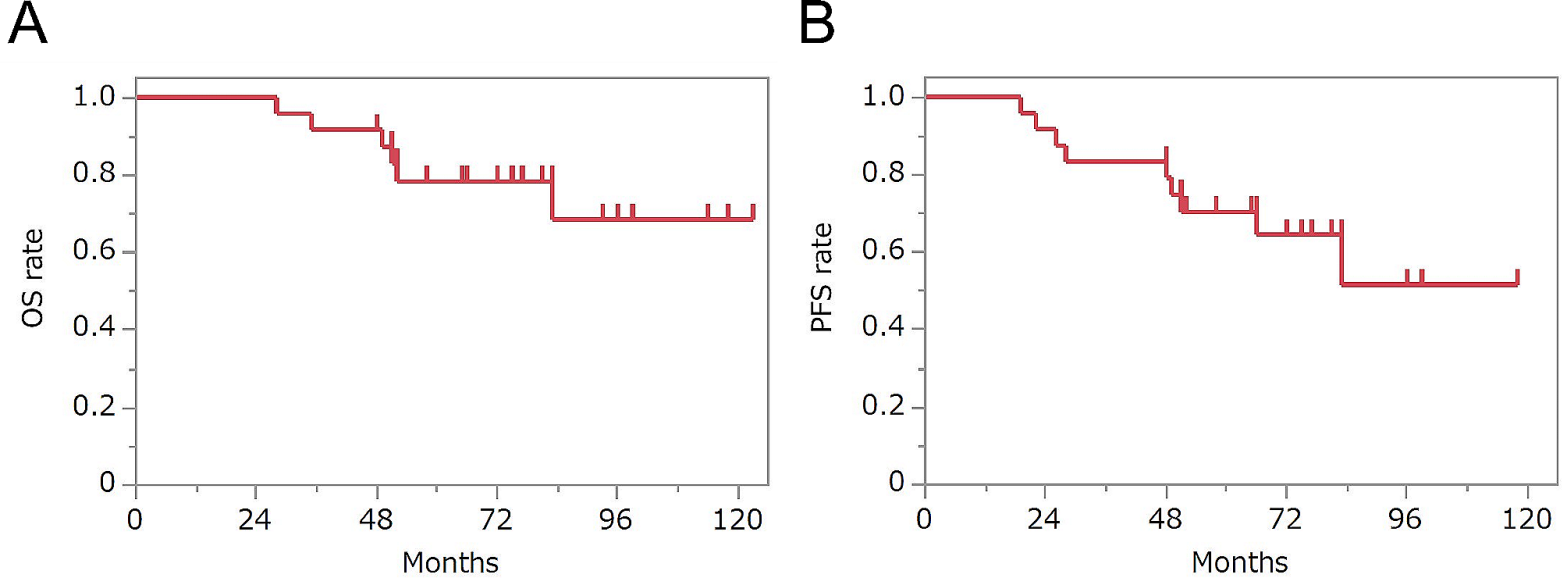
Overall (**A**) and progression-free (**B**) survival curves of patients who received additional chemotherapy for esophageal cancer after near-circumferential or full-circumferential noncurative endoscopic submucosal dissection

The patient and tumor characteristics are summarized in Table 1. The median follow-up period was 74 months (range: 28–123 months). The 3-year and 5-year OS rates were 92% and 78%, respectively, while the 3-year and 5-year PFS rates were 83% and 70%, respectively (Fig. 2). Our univariate analyses revealed that there was no statistically significant risk factor for OS or PFS (Table 2).

**Table 1:** Patient and tumor characteristics

	*N* (%)
Sex	
Male	20 (83)
Female	4 (17)
Median age (years; range)	70 (55–81)
Location of tumor	
Cervical	1 (4)
Upper thoracic	2 (8)
Middle thoracic	12 (50)
Lower thoracic or abdominal	9 (38)
Median diameter of tumor (mm; range)	32 (7–59)
Pathological invasion depths	
MM	11 (46)
SM1	5 (21)
SM2	8 (33)
Resection margin status	
Negative	20 (83)
Positive	4 (17)
LVI	
No	5 (21)
Yes	19 (79)

MM: muscularis mucosae; SM: submucosal layer; LVI: lymphovascular invasion

**Table 2:** Univariate analyses of OS and PFS

	*N*	Five-year OS		Five-year PFS	
		%	*p* value	%	*p* value
Sex					
Male	20	84	0.22	75	0.78
Female	4	50		50	
Age (years)					
< 70	12	92	0.23	75	0.95
≥ 70	12	65		66	
Location of tumor					
Cervical or Upper thoracic	3	100	0.32	100	0.20
Middle thoracic, Lower thoracic, or abdominal	21	75		66	
Diameter of tumor (mm)					
< 30	11	90	0.15	90	0.09
≥ 30	13	69		54	
Pathological invasion depths					
MM	11	70	0.88	52	0.08
SM	13	85		85	
Resection margin status					
Negative	20	73	0.74	69	0.94
Positive	4	100		75	
LVI					
No	5	80	0.90	80	0.42
Yes	19	78		68	

OS: overall survival; PFS: progression-free survival; MM: muscularis mucosae; SM: submucosal layer; LVI: lymphovascular invasion

#### Failure patterns and salvage treatments

Progression was detected in 4 patients (local recurrence in 1, lymph node metastasis in 2, and distant metastasis in 1). Those who experienced local recurrence 25 months after the completion of additional CRT underwent salvage argon plasma coagulation and stayed alive thereafter without experiencing recurrence anymore. Among the 2 cases of lymph node metastasis, one patient presented an in-field metastasis 18 months after the completion of additional CRT and received salvage chemotherapy, achieving disease control and currently remaining alive. The other demonstrated an out-of-field metastasis 47 months after the completion of additional CRT and underwent salvage CRT, achieving disease control but dying 20 months after the initiation of salvage CRT due to acute myeloid leukemia (AML). The patient who had distant metastases in the lungs 66 months after the completion of additional CRT underwent surgery through which he achieved disease control and remained alive thereafter.

### Toxicity

Grade 2 esophageal stenosis occurred in 8 (33%) patients. No Grade 3 or worse esophageal stenosis was detected. Of the 8 patients with Grade 2 stenosis, 4 (17%) patients initially presented Grade 2 esophageal stenosis before additional CRT and stenosis persisted after the completion of additional CRT (Table 3). These patients underwent balloon and/or bougie dilatation. Three of the 4 patients improved to Grade 1 or lower within 5 months from additional CRT, while the other patient still requires regular bougie dilatation 8 years after the completion of additional CRT. The remaining 4 (17%) patients developed *de novo* stenosis within 5 months after the completion of additional CRT (Table 4). These patients also underwent balloon dilation and/or bougie dilatation. Three of the 4 patients improved to Grade 1 or lower within 3 months from the initial dilatation for esophageal stenosis, whereas this improvement required 18 months in the last patient. Table 5 summarizes the acute and late toxicity other than esophageal stenosis. Grade 3 or worse acute toxicity, including anemia, neutropenia, thrombocytopenia, and esophagitis occurred in 1 (4%), 7 (29%), 1 (4%), and 1 (4%) patients, respectively. None of these patients had symptomatic radiation pneumonitis. Late toxicity of Grade 2 pericardial effusion was observed in 3 (13%) patients; however, no Grade 3 or worse pericardial effusion was observed. As described above, one patient (4%) developed AML and died, which was recorded as a Grade 5 late toxicity of secondary malignancy because we could not rule out the relationship between AML and additional CRT.

**Table 3:** Patients with Grade 2 esophageal stenosis before additional chemoradiotherapy

ESD circumference	Tumor location	Tumor diameter (mm)	Pathological invasion depths	Resection margin status	LVI	Time required for improvement after CRT (months)
full	Middle thoracic	57	pT1a-MM	Negative	Yes	4
7/8	Lower thoracic	24	pT1b-SM2	Negative	Yes	5
full	Cervical	30	pT1b-SM2	Negative	No	5
full	Middle thoracic	40	pT1b-SM1	Positive	No	No improvement

ESD: endoscopic submucosal dissection; LVI: lymphovascular invasion; CRT: chemoradiotherapy; MM: muscularis mucosae; SM: submucosal layer

**Table 4:** Patients with *de novo* Grade 2 esophageal stenosis after additional chemoradiotherapy

ESD circumference	Tumor location	Tumor diameter (mm)	Pathological invasion depths	Resection margin status	LVI	Time required for development after CRT completion (months)	Time-lapse from initial dilatation to improvement (months)
9/10	Lower thoracic	55	pT1a-MM	Positive	Yes	1	2
full	Lower thoracic	38	pT1a-MM	Positive	No	5	18
full	Middle thoracic	59	pT1a-MM	Negative	Yes	5	1
4/5	Middle thoracic	21	pT1a-MM	Negative	No	2	3

ESD: endoscopic submucosal dissection; LVI: lymphovascular invasion; CRT: chemoradiotherapy; MM: muscularis mucosae

**Table 5:** Toxicity other than esophageal stenosis

Toxicity	*N* (%)			
	Grade 2	Grade 3	Grade 4	Grade 5
*Acute*				
Anemia	4 (17)	1 (4)	0 (0)	0 (0)
Neutropenia	8 (33)	6 (25)	1 (4)	0 (0)
Thrombocytopenia	5 (21)	1 (4)	0 (0)	0 (0)
Dermatitis	4 (17)	0 (0)	0 (0)	0 (0)
Esophagitis	4 (17)	1 (4)	0 (0)	0 (0)
Renal function	1 (4)	0 (0)	0 (0)	0 (0)
*Late*				
Pericardial effusion	3 (13)	0 (0)	0 (0)	0 (0)
Secondary AML	0	0	0	1 (4)

AML: Acute myeloid leukemia

## Discussion

Some groups have retrospectively reported on the outcomes of additional CRT after noncurative endoscopic resection. Nishibuchi et al. [11] retrospectively reviewed 37 patients with superficial esophageal cancer who received additional RT with or without chemotherapy after noncurative endoscopic resection. The circumference of the tumor was < 3/4 in 25 (68%) patients, ≥ 3/4 in 8 (22%) patients, and unknown in 4 (11%) patients. ENI was performed with 40–48 Gy followed by either an external-beam RT boost with a total median dose of 60 Gy in 30 fractions or an intraluminal brachytherapy boost with an RT dose of 10 Gy in four fractions. Twenty-five patients received concurrent chemotherapy with a combination of platinum-based drugs and 5-FU. The reported 5-year OS and PFS rates were 78% and 64%, respectively. Hisano et al. [12] retrospectively analyzed 13 superficial esophageal cancer patients who underwent additional RT with or without chemotherapy after noncurative ESD. Of the 13 patients, 6 (46%) with mucosal defects involving ≥ 3/4 of the esophageal circumference after ESD were included. All patients received ENI with RT doses of 40–41.4 Gy in 20–23 fractions. Among them, 8 patients received BI. One patient with positive resection margins received a total RT dose of 61.4 Gy, while the remaining 7 received total RT doses of 50–50.4 Gy. Concurrent chemotherapy (a combination of CDDP and 5-FU or S-1 alone) was administered to 4 patients. They showed that the 3-year OS and cause-specific survival were 67% and 82%, respectively. Even though our study only included esophageal SCC patients with mucosal defects affecting ≥ 3/4 of the esophageal circumference after noncurative ESD, which might have a poorer prognosis than those with limited mucosal defects, our results were similar to theirs. These findings suggested that additional CRT is effective even in patients who have undergone near-circumferential or full-circumferential noncurative ESD.

Some reports have demonstrated that survival outcomes of additional CRT after ESD are comparable to those of additional esophagectomy. Tanaka et al. [13] retrospectively investigated the outcomes of 52 patients who underwent ESD for superficial esophageal cancer with SM invasion. Of the 52 patients, 33 received additional CRT, while 19 underwent additional esophagectomy after ESD. The 5-year OS and PFS rates for the additional CRT group were 80% and 70%, and those for the additional esophagectomy group were 90% and 90%, respectively. No significant differences were observed between the groups in terms of both OS (*p* = 0.17) and PFS (*p* = 0.13). Suzuki et al. [1] retrospectively reviewed 60 patients who underwent additional treatment after noncurative ESD for superficial esophageal cancer with positive resection margin or LVI or SM invasion. Thirty-four patients received additional CRT, while 26 patients underwent additional esophagectomy. The 4-year OS and PFS rates for the additional CRT group were 84% and 65%, and those for the additional esophagectomy groups were 92% and 73%, respectively. No significant differences were observed between the groups in terms of both OS (*p* = 0.87) and PFS (*p* = 0.41). Meanwhile, the mortality of additional esophagectomy was not negligible; similar to conventional esophagectomy, the mortality rate of additional esophagectomy after ESD has been reported as approximately 5% [12–15]. On the contrary, the reported Grade 5 acute toxicity of additional CRT was zero [7, 11, 12, 17]. These findings suggest that additional CRT is an alternative treatment option to additional esophagectomy after noncurative ESD and it could be valuable as a part of the esophagus-preserving strategy in patients with superficial esophageal cancer.

Previous reports suggested that the combination of near-circumferential or full-circumferential ESD and additional CRT is associated with a certain frequency of esophageal stenosis [11, 12]. As described above, Nishibuchi et al. [11] performed additional RT with or without chemotherapy after noncurative endoscopic resection in 37 patients with superficial esophageal cancer. They reported that 6/8 (75%) patients with tumor circumferences ≥ 3/4 developed Grade 2 or worse esophageal stenosis, while no patients with tumor circumference < 3/4 experienced this complication. Tumor circumference ≥ 3/4 was significantly associated with stenosis (*p* < 0.001). Similarly, as described above, Hisano et al. [11] performed additional RT with or without chemotherapy after noncurative ESD in 13 patients with superficial esophageal cancer. They reported that 2 of 6 (33%) patients with mucosal defects occupying ≥ 3/4 of the esophageal circumference developed Grade 2 or worse esophageal stenosis, whereas none of the patients with < 3/4 circumference had such clinical manifestations. Our results of esophageal stenosis were consistent with their reports. The development of *de novo* esophageal stenosis in some patients after additional CRT may be mainly attributed to the combined effects of mucosal damage caused by ESD itself and fibrosis due to the healing process of ESD mucosal damage [18]. Furthermore, additional CRT may lead to esophageal stenosis. Late adverse effects of esophageal stenosis due to CRT-induced fibrosis have been reported in patients with esophageal cancer even if they do not undergo ESD [19]. In our study, two patients developed *de novo* esophageal stenosis five months after the completion of additional CRT. Although we cannot clarify the degree of the effect, additional CRT could have a certain effect on the esophageal stenosis of these patients. Our results suggest that an improvement in esophageal stenosis is obtained over time with balloon and/or bougie dilation, with only one patient experiencing refractory esophageal stenosis. This patient had undergone full-circumferential ESD and received BI owing to the presence of positive margins, which may have contributed to the development of refractory stenosis. The extensive mucosal defect from full-circumferential ESD, combined with the increased radiation dose to the esophageal mucosa from BI potentially promoting radiation-induced fibrosis, might have increased the risk of developing refractory stenosis in this patient. However, despite mild dysphagia, very little esophageal stenosis was observed on endoscopy, and the patient maintained a satisfactory dietary intake. The number of patients requiring long-term management is minimal, and esophageal stenosis that occurs as a result of additional CRT is manageable.

Grade 3 or worse esophagitis has been reported in 4–13% of patients [7, 11, 12, 17]. Our rate of esophagitis was within the reported results, and the esophagitis could be medically managed. Regarding hematological toxicity, anemia, neutropenia, and thrombocytopenia are mainly caused by chemotherapy. In this study, the rate of such toxicity was similar to that reported in previous studies [7, 11, 12, 17], and this toxicity could also be medically controlled.

We could not determine whether the initial CRT, salvage CRT, or other factors yielded AML in our patients. However, there have been some reports of secondary AML observed after definitive CRT for esophageal cancer [20, 21]. Nishimura et al. [21] reported the long-term follow-up results of a randomized Phase II trial that compared short-term infusion with protracted CDDP and 5-FU infusion for concurrent CRT in patients with esophageal cancer. Of the 91 patients who underwent definitive CRT, 1 (1%) developed AML (which was defined as Grade 5 late toxicity) and died. Although secondary malignancy is not common in patients who undergo CRT, radiation oncologists should be mindful of this potential risk.

The main limitation of the study is that it is a single-center retrospective study with a small study sample. We cannot exclude the potential bias of physicians on the indication of additional CRT and surgery after ESD in our institution.

## Conclusion

Additional CRT is a viable treatment option even in patients who have undergone near-circumferential or full-circumferential noncurative ESD as well as those with limited tumors. Esophageal stenosis after additional CRT following near-circumferential or full-circumferential noncurative ESD is manageable and acceptable.

## Data Availability

No datasets were generated or analysed during the current study.

## Abbreviations

ESD: Endoscopic submucosal dissection
CRT: Chemoradiotherapy
RT: Radiotherapy
ENI: Elective nodal irradiation
BI: Boost irradiation
MM: Muscularis mucosae
SM: Submucosal layer
LVI: Lymphovascular invasion
SCC: Squamous cell carcinoma
CTV: Clinical target volume
PTV: Planning target volumes
3D-CRT: Three-dimensional conformal radiotherapy
CDDP: Cisplatin
NDP: Nedaplatin
5-FU: 5-fluorouracil^18^F-FDG: [^18^F] fluorodeoxyglucose
PET: Positron emission tomography
OS: Overall survival
PFS: Progression-free survival
AML: Acute myeloid leukemia

## References

[1] Januszewicz W, Fitzgerald RC. Early detection and therapeutics. Mol Oncol. 2019;13:599–613. 30677217 10.1002/1878-0261.12458 PMC6396365

[2] Yokoyama A, Ohmori T, Makuuchi H, Maruyama K, Okuyama K, Takahashi H, et al. Successful screening for early esophageal cancer in alcoholics using endoscopy and mucosa iodine staining. Cancer. 1995;76:928 − 34.3. 8625217 10.1002/1097-0142(19950915)76:6<928::AID-CNCR2820760604>3.0.CO;2-5

[3] Bollschweiler E, Baldus SE, Schröder W, Prenzel K, Gutschow C, Schneider PM, et al. High rate of lymph-node metastasis in submucosal esophageal squamous-cell carcinomas and adenocarcinomas. Endoscopy. 2006;38:149 − 56. 16479422 10.1055/s-2006-924993

[4] Eguchi T, Nakanishi Y, Shimoda T, Iwasaki M, Igaki H, Tachimori Y, et al. Histopathological criteria for additional treatment after endoscopic mucosal resection for esophageal cancer: analysis of 464 surgically resected cases. Mod Pathol. 2006;19:475–80. 16444191 10.1038/modpathol.3800557

[5] Hatta W, Koike T, Uno K, Asano N, Masamune A. Management of superficial esophageal squamous cell carcinoma and early gastric cancer following non-curative endoscopic resection. Cancers (Basel). 2022;14:3757. 35954421 10.3390/cancers14153757 PMC9367302

[6] Watanabe M, Otake R, Kozuki R, Toihata T, Takahashi K, Okamura A, et al. Recent progress in multidisciplinary treatment for patients with esophageal cancer. Surg Today. 2020;50:12–20. 31535225 10.1007/s00595-019-01878-7 PMC6952324

[7] Minashi K, Nihei K, Mizusawa J, Takizawa K, Yano T, Ezoe Y, et al. Efficacy of endoscopic resection and selective chemoradiotherapy for Stage I esophageal squamous cell carcinoma. Gastroenterology. 2019;157:382–e3903. 31014996 10.1053/j.gastro.2019.04.017

[8] Jonas E, Sjöqvist S, Elbe P, Kanai N, Enger J, Haas SL, et al. Transplantation of tissue-engineered cell sheets for stricture prevention after endoscopic submucosal dissection of the oesophagus. U Eur Gastroenterol J. 2016;4:741–53. 10.1177/2050640616631205 PMC5386228 28408991

[9] Toya R, Matsuyama T, Saito T, Imuta M, Shiraishi S, Fukugawa Y, et al. Impact of hybrid FDG-PET/CT on gross tumor volume definition of cervical esophageal cancer: reducing interobserver variation. J Radiat Res. 2019;60:348–52. 30864652 10.1093/jrr/rrz004 PMC6530614

[10] Uno K, Koike T, Kusaka G, Takahashi Y, Ara N, Shimosegawa T. Risk of metachronous recurrence after endoscopic submucosal dissection of esophageal squamous cell carcinoma. Dis Esophagus. 2017;30:1–8. 28475742 10.1093/dote/dox005

[11] Nishibuchi I, Murakami Y, Adachi Y, Imano N, Takeuchi Y, Tkahashi I, et al. Effectiveness of salvage radiotherapy for superficial esophageal cancer after non-curative endoscopic resection. Radiat Oncol. 2020;15:133. 32487186 10.1186/s13014-020-01582-8 PMC7268314

[12] Hisano O, Nonoshita T, Hirata H, Sasaki T, Watanabe H, Wakiyama H, et al. Additional radiotherapy following endoscopic submucosal dissection for T1a-MM/T1b-SM esophageal squamous cell carcinoma improves locoregional control. Radiat Oncol. 2018;13:14. 29378603 10.1186/s13014-018-0960-y PMC5789550

[13] Tanaka T, Ueno M, Iizuka T, Hoteya S, Haruta S, Udagawa H. Comparison of long-term outcomes between esophagectomy and chemoradiotherapy after endoscopic resection of submucosal esophageal squamous cell carcinoma. Dis Esophagus. 2019;32:1–8. 10.1093/dote/doz023 30980070

[14] Suzuki G, Yamazaki H, Aibe N, Masui K, Kimoto T, Nagasawa S, et al. Chemoradiation versus surgery for superficial esophageal squamous cell carcinoma after noncurative endoscopic submucosal dissection: comparison of long-term oncologic outcomes. Radiat Oncol. 2022;17:191. 36401267 10.1186/s13014-022-02162-8 PMC9675257

[15] Linden PA, Towe CW, Watson TJ, Low DE, Cassivi SD, Grau-Sepulveda M, et al. Mortality after esophagectomy: analysis of individual complications and their association with mortality. J Gastrointest Surg. 2020;24:1948–54. 31410819 10.1007/s11605-019-04346-2

[16] Oesophago-Gastric Anastomotic Audit (OGAA) Collaborative: Writing Committee, Committee S, Leads N, Leads S. Collaborators. Mortality from esophagectomy for esophageal cancer across low, middle, and high-income countries: an international cohort study. Eur J Surg Oncol. 2021;47:1481-8. 10.1016/j.ejso.2020.12.006 33451919

[17] Kawaguchi G, Sasamoto R, Abe E, Ohta A, Sato H, Tanaka K, et al. The effectiveness of endoscopic submucosal dissection followed by chemoradiotherapy for superficial esophageal cancer. Radiat Oncol. 2015;10:31. 25636830 10.1186/s13014-015-0337-4 PMC4316795

[18] Yu M, Tan Y, Liu D. Strategies to prevent stricture after esophageal endoscopic submucosal dissection. Ann Transl Med. 2019;7:271. 31355238 10.21037/atm.2019.05.45 PMC6614329

[19] Kato K, Nakajima TE, Ito Y, Katada C, Ishiyama H, Tokunaga S, et al. Phase II study of concurrent chemoradiotherapy at the dose of 50.4 gy with elective nodal irradiation for stage II-III esophageal carcinoma. Jpn J Clin Oncol. 2013;43:608–15. 23585687 10.1093/jjco/hyt048

[20] Hiraoka S, Sakanaka K, Iwai T, Fujii K, Inoo H, Mizowaki T. Therapy-related acute myeloid leukemia 2 months after chemoradiotherapy for esophageal cancer: a case report. Case Rep Oncol. 2020;13:299–303. 32308595 10.1159/000506449 PMC7154239

[21] Nishimura Y, Hiraoka M, Koike R, Nakamatsu K, Itasaka S, Kawamura M, et al. Long-term follow-up of a randomized phase II study of cisplatin/5-FU concurrent chemoradiotherapy for esophageal cancer (KROSG0101/JROSG021). Jpn J Clin Oncol. 2012;42:807–12. 22811410 10.1093/jjco/hys112

